# Highly selective skeletal isomerization of cyclohexene over zeolite-based catalysts for high-purity methylcyclopentene production

**DOI:** 10.1038/s42004-021-00472-8

**Published:** 2021-03-11

**Authors:** Hao Xu, Zhaofei Li, Shijun Meng, Jack Jarvis, Hua Song

**Affiliations:** Department of Chemical and Petroleum Engineering, University of Calgary, Calgary, AB Canada

**Keywords:** Heterogeneous catalysis, Porous materials

## Abstract

Cyclohexene skeletal isomerization towards methylcyclopentene is an economically favorable process due to the higher added value of the product. Traditional oxide-based catalysts face the challenge of achieving both high activity and stability. In this work, cyclohexene skeletal isomerization is achieved under mild conditions over designed zeolite-based catalysts with 96.8 wt.% liquid yield, 95.8 wt.% selectivity towards methylcyclopentene and satisfactory stability for multiple runs. The favorable performance is attributed to the unique acidic, structural and morphological features of the optimized cobalt/NaUZSM-5 catalyst. Further experimental data and DFT studies suggest that a carboncationic mechanism might be followed and that the reaction mainly occurs within the internal pores of the zeolite structures.

## Abstract (web summary)

Cyclohexane skeletal isomerisation is an important catalytic reaction, but generally involves a trade-off between catalyst activity and selectivity. Here zeolite-based catalysts improve the selectivity of this reaction towards methylcyclopentene while retaining good activity, attributed to the acidic, structural and morphological features of the optimized catalysts.

## Introduction

Cyclohexene is one of the most common products of the light oil pyrolysis process, which can be directly produced by pyrolysis of rice straw^1^. Many reactions may take place during the catalytic upgrading process including ring opening, cracking, isomerization, alkylation, aromatization, and hydrogenation. It has been reported that the products of cyclohexene conversion include various types of hydrocarbons with a wide range of carbon numbers, from light gases such as ethylene, propane, propylene, and 1,3-butadiene, to liquid products including cyclohexane, cyclohexadiene, and benzene, as well as heavy polyaromatic hydrocarbons towards coke formation^2–4^. Due to the inherent complexity of the involved reactions, high product selectivity is often hard to achieve^5^. Therefore, the establishment of a strategy to get much better control of the catalytic cyclohexene conversion process, namely improving the selectivity towards specific desired high-value products, is of great significance.

Methylcyclopentene (mcp) has the same chemical formula (C_6_H_10_) as cyclohexene, and can be produced from cyclohexene through a skeletal isomerization reaction^6,7^. It is specifically valuable for the synthesis of a series of chemical derivatives with great demand from petrochemical plants^8^. For example, it can be co-polymerized with ethylene to form polyolefins with unique properties such as high mechanical strength, corrosion resistance, and electrical conductivity, while negligible incorporation is observed when cyclohexene is used as the monomer feed^9,10^. Besides, it is also used for the synthesis of various insecticides, resin intermediates, and related products^8^. The industrial route for producing methylcyclopentene mainly relies on the dehydrogenation of methylcyclopentane, but the activities of catalysts are invariably too low for efficient production, and also methylcyclopentane is relatively expensive compared to cyclohexene^8^. Moreover, benzene is also widely obtained as an unfavorable by-product, and the subsequent separation process causes increased capital cost and operational complexity^8^. Therefore, the price of methylcyclopentene, especially 1-methylcyclopentene due to its tertiary cyclic olefin structure, is much higher than its other isomers such as cyclohexene^11^. It would be highly profitable if methylcyclopentene with high purity could be produced through a facile process from readily available sources, and the selective conversion of cyclohexene towards methylcyclopentene through skeletal isomerization reaction under mild conditions is thus economically attractive and deserves focused investigation.

The traditional catalysts for cyclohexene skeletal isomerization are mainly based on metal oxides including silica, alumina, zirconia, titania, or their combinations. It is widely observed that the activity of catalyst is positively correlated to increasing surface acidity^6,12–14^. However, highly active catalysts with high surface acidity can promote a series of side reactions such as hydrogen transfer, cracking, and coking, lowering the selectivity towards methylcyclopentene. On the other hand, catalysts with low surface acidity demonstrate insufficient activity for efficient methylcyclopentene production^13,15,16^. A catalyst with both high activity and methylcyclopentene selectivity still remains unreported to our knowledge.

In this work, a series of zeolite-based catalysts are designed for promoting cyclohexene conversion under industrially favorable mild conditions of 400 °C and near atmospheric pressure. A set of commonly used metal-oxide-based catalysts are also used as references for better comparison. A wide variety of characterization tools including micro-GC (micro-chromatography), GC-MS (gas chromatography-mass spectrometry), TGA (thermalgravimetric analysis), N_2_ physisorption, NH_3_-TPD (ammonia-temperature programmed desorption), DRIFTS (diffuse reflectance infrared Fourier transform spectroscopy), ICP-OES (inductively coupled plasma-optical emission spectrometry), SEM (scanning electron microscope) and XRD (X-ray diffraction), coupled with DFT (density functional theory) calculations are used to explore the property-performance relationship of the charged catalysts. Based on these results an optimization strategy is developed and implemented to guide catalyst design and synthesis for achieving maximized selectivity of methylcyclopentene with acceptable cyclohexene conversion.

## Results and discussion

### Comparison of UZSM-5 and metal-oxide catalysts

**Fig. 1:**
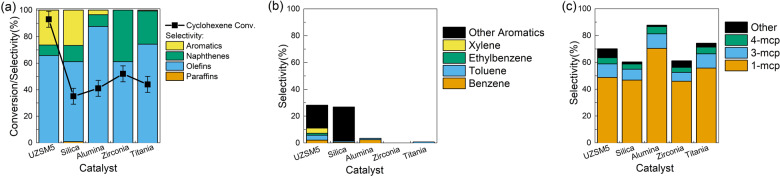
Performances of UZSM-5 and metal oxides for catalytic cyclohexene conversion. **a** Cyclohexene conversion and PONA selectivity. **b** Selectivity of aromatic species. **c** Selectivity of olefin species. The error bars represent the standard deviation of cyclohexene conversion measurement.

First, a home-made zeolite-based catalyst (UZSM-5) was prepared with optimized synthesis conditions so that the morphology can be better controlled to be more uniform and with a cylindrical shape. This catalyst was compared with various conventional metal-oxide-based catalysts for catalytic cyclohexene conversion. The performances of these catalysts are shown in Fig. 1 and Table S2, and detailed compositions of liquid products are listed in Table S10. According to Fig. 1a UZSM-5 demonstrates much higher cyclohexene conversion and similar olefin selectivity compared with traditional oxide catalysts, proving its potential for catalytic conversion of cyclohexene to valuable products. However, the selectivity towards aromatics is also the highest. Detailed analysis of generated aromatic products in Fig. 1b indicates the type of aromatics varies with different catalysts. UZSM-5 leads to the formation of BTEX (benzene, toluene, ethylbenzene, and xylenes) family, and other aromatics including monocyclic species with 9–11 carbon atoms such as mesitylene and limited number of bicyclic species such as naphthalene are also detected. This can be attributed to the primary function of zeolite-based catalyst for aromatization, which is widely reported and utilized in industry^5,17^. Moreover, silica leads to a unique distribution of aromatic products, which mainly consists polycyclic aromatic hydrocarbons with 2 or 3 rings rather than BTEX species. Alumina and titania result in limited production of benzene and toluene, while zirconia demonstrates no aromatic selectivity. The varied aromatic formation characteristic is well understood by the shape-selective effect of catalysts with different pore structures, as indicated in Fig. S5. However, despite the dramatic difference in aromatic species, it is indicated in Fig. 1c that the main olefin products formed overall catalysts are three isomers of methylcyclopentene, implying that the skeletal isomerization of cyclohexene can be well observed regardless of the charged catalyst structures. To the best of our knowledge, the favorable catalytic performances of cyclohexene isomerization over zeolite-based catalyst have not been reported and are thus worth further investigation.

### Catalytic performances of zeolite-based catalysts

**Fig. 2:**
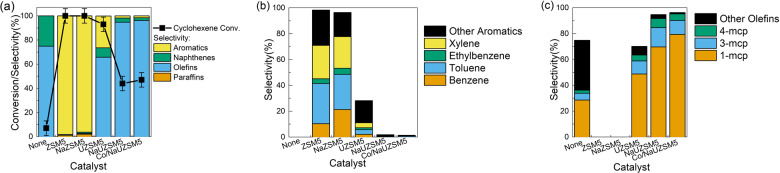
Performances of zeolite-based catalysts for catalytic cyclohexene conversion. **a** Cyclohexene conversion and PONA selectivity. **b** Selectivity of aromatic species. **c** Selectivity of olefin species. The error bars represent the standard deviation of cyclohexene conversion measurement.

In order to get further understanding of catalytic cyclohexene conversion with zeolite-based catalysts, a series of zeolite catalysts were synthesized, and their corresponding performances are compared in Fig. 2 and Table S2. Detailed information of zeolite supports and corresponding naming policy are shown in the “Methods”. According to Fig. 2a, it is evident that zeolite catalysts show entirely different performances compared to the pure thermal pyrolysis process without catalyst in terms of cyclohexene conversion and product selectivity. Detailed analysis of aromatic species in Fig. 2b suggests BTEX family is the main product over ZSM-5 and NaZSM-5 catalysts, but their formation is greatly inhibited over UZSM-5 series catalysts. Figure 2c witnesses that although no olefin is formed over ZSM-5 and NaZSM-5 catalysts, methylcyclopentene remains as the main olefin product when UZSM-5 series catalysts are engaged, implying the unique catalytic performances of UZSM-5 supported catalyst compared to conventional ZSM-5 based ones. Comparing UZSM-5 based catalysts, it can be concluded that ion exchange by Na^+^ and metal loading of cobalt further enhance the selectivity towards methylcyclopentene formation. It is worth noting that over 95 wt.% methylcyclopentene selectivity is successfully achieved over Co/NaUZSM-5 catalyst, which is much higher than the value over oxide-based catalysts in Fig. 1. This favorable performance is highly promising for the production of methylcyclopentene with high purity through a simple process, which demonstrates great potential for industrial application with high profitability.

### Stability test

**Fig. 3:**
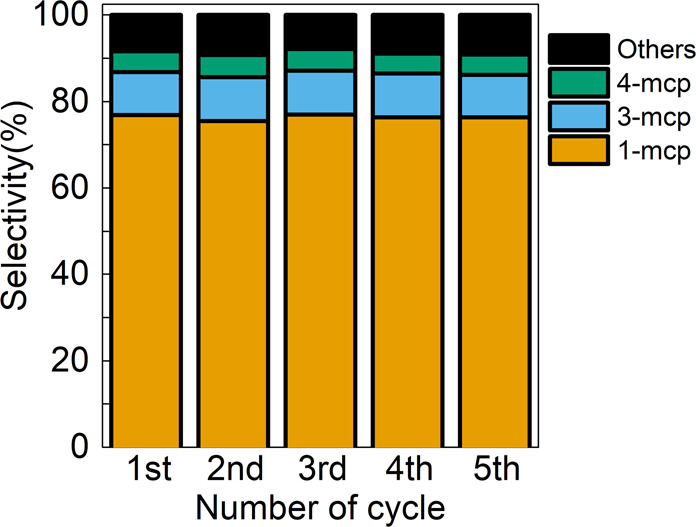
Catalyst stability test. Catalytic performance of Co/NaUZSM-5 in five reaction cycles.

In order to evaluate the stability of highly selective cyclohexene skeletal isomerization catalyst, the spent Co/NaUZSM-5 was collected after the reaction and directly used in the following runs without further treatment for five consecutive cycles. The results are shown in Fig. 3. It can be clearly seen that the selectivity towards desired product methylcyclopentene remains almost unchanged, strongly indicating the satisfactory stability of the catalyst in multiple runs.

### Relationship between surface acidity and catalytic performances of zeolite-based catalysts

**Fig. 4:**
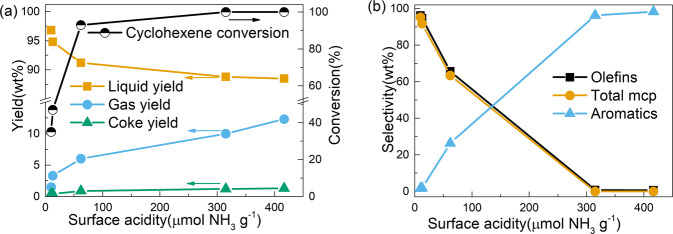
Relationship between catalytic performance and the surface acidity of catalysts. **a** Overall analysis. **b** Selectivity of olefin, methylcyclopentene, and aromatics.

From Fig. 2a, dramatically different performances of zeolite-based catalyst can be witnessed and determination of the reason behind is of great importance. It is well known that the number of acid sites plays an important role on catalytic performances of zeolite-based catalysts^18^. Therefore, the number of acid sites in different catalysts was first quantified by NH_3_-TPD, which is pre-calibrated by standard ZSM-5 zeolite support (Fig. S6 and Table S4). The different surface acidities are further confirmed by in situ pyridine adsorption via DRIFTS analyses (Fig. S7). Then, catalytic performance of zeolite-based catalysts was correlated with the number of acid sites and the results are shown in Fig. 4. According to Fig. 4a, the overall catalytic performance of different catalysts demonstrates a clear trend along with increasing number of acid sites: cyclohexene conversion as well as gas and coke yield go up, and the liquid yield decreases correspondingly. This trend can be explained by the strong capability of cyclohexene adsorption on the acidic sites, which triggers a series of reactions including cracking and coking. The gas composition analysis in Table S1 also indicates hydrocarbon species with 1–4 carbon numbers are formed as the main products of the cracking process, which is also consistent with literature report^2,5^. Besides, the selectivity towards aromatics, olefin, and desired product methylcyclopentene also follows a monotonous trend along with increasing number of acid sites (Fig. 4b). The positive correlation between aromatic selectivity and the number of acid sites suggests that higher acidity is favorable for aromatic formation, which is widely accepted as the main function of zeolite catalysts^18–22^. However, with limited acidity, the aromatic content can be greatly lowered and olefin products with high purity of methylcyclopentene predominates, indicating that aromatization and isomerization are two competing reactions in the catalytic cyclohexene conversion process, which can be effectively controlled by tuning the number of acid sites.

Nevertheless, since the silica to alumina ratio is maintained at 80:1 in both ZSM-5 and UZSM-5 catalyst, the reason why the number of acid sites is completely different needs to be fully investigated. According to ICP-OES results in Table S5, the diverse acidity of zeolite catalysts can be attributed to the substitution of acid sites by Na or K, since higher content of Na and K leads to lower number of acid sites. Further calculation indicates the amount of Na^+^ in NaZSM-5 is equivalent to 33% of theoretical H^+^ amount in ZSM-5, implying the ion exchange process is relatively insufficient. In contrast, UZSM-5 contains a considerable amount of K^+^, which equals 75% of theoretical H^+^ amount. The high potassium content is further proved to be from the template solution TPAOH (Table S5). Interestingly, further ion exchange of UZSM-5 by NaOH leads to both high Na and K contents, and the total amount is even higher (160%) than theoretical H^+^ amount, indicating K is present not only on acid sites but also as K salt in the pore structure of UZSM-5. In order to further understand the presence of K on the structural properties of zeolite catalysts, N_2_ adsorption/desorption was carried out (Fig. S4 and Table S3). The results indicate that zeolite-based catalysts are all microporous materials and the average pore sizes fall in the range of 0.55–0.60 nm, which is highly distinguishable from oxide-based mesoporous materials (Fig. S5). However, the average pore size of UZSM-5 is larger than that of ZSM-5, accompanied by slightly increased micropore volume and surface area, indicating the presence of K in the zeolitic pores leads to a non-negligible enhancement of these structural properties. This is also confirmed by the unit cell parameters calculated from the diffraction patterns (Fig. S8 and Table S6). Moreover, it is noted that the FWHM (full width at half maximum) of diffraction peaks are clearly different for ZSM-5 (0.27°) and UZSM-5 (0.41°). The increase of FWHM could be due to smaller particle size of UZSM-5 and this conclusion is also confirmed by SEM images as shown in Fig. S9, since more uniformly shaped fine particles with clearly smaller dimensions are observed for UZSM-5 compared to ZSM-5. Based on these observations, the unique catalytic performances of UZSM-5 can be better explained by the change of surface and structural features, where the presence of K plays a major role. On the one hand, K lowers the number of acid sites through direct H^+^ substitution, which is already well documented^23^. On the other hand, the presence of K in the zeolite framework structure also leads to enlarged micropores of zeolite catalysts, which is also reported in previous studies^24,25^. Then, a more efficient ion exchange process can be realized and the acidity can be more effectively controlled, which is proved by high Na content in NaUZSM-5 and corresponding low acidity. It can be concluded that the changes of acidity and structure caused by K introduction make combined contributions to the favorable methylcyclopentene selectivity over zeolite-based catalysts. Moreover, the uniform morphology of UZSM-5 could also be beneficial for the high selectivity of methylcyclopentene. However, this preferred morphology can be more likely linked to the optimized methods and conditions for zeolite synthesis rather than K introduction, which is evidenced by the SEM images of K-free UZSM-5 catalysts (Fig. S10).

### Mechanism interpretation

**Fig. 5:**
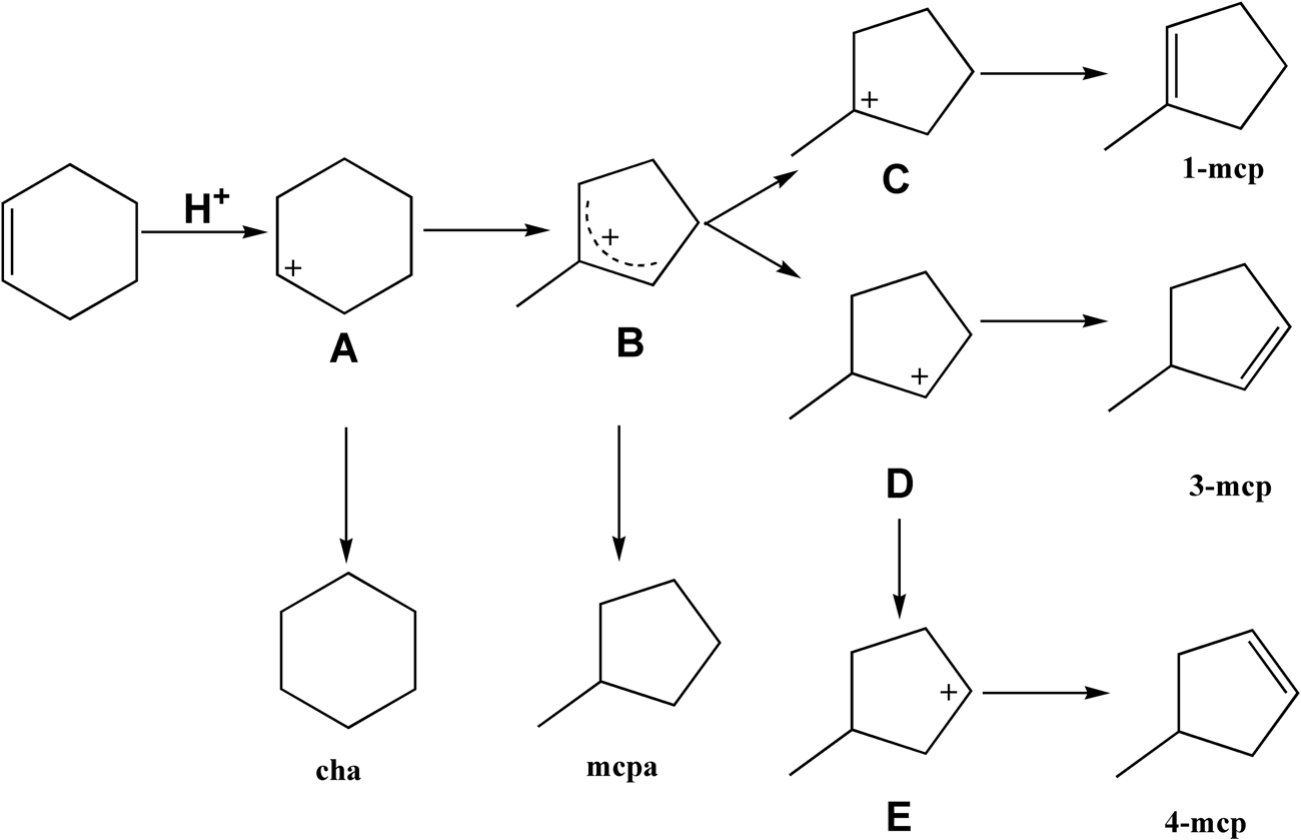
Proposed mechanism. Carboncationic mechanism of cyclohexene skeletal isomerization.

In previous studies, cyclohexene skeletal isomerization is reported to proceed through a carboncationic (carbenium) mechanism as shown in Fig. 5^6^. First, cyclohexene can be selectively adsorbed through the C = C double bond on the catalytic surface, particularly on the Bronsted acid sites. Then, the Bronsted acid sites act as the proton donor and the cyclohexene get protonated to form a carboncationic intermediate (**A**). Next, the carboncationic intermediate of cyclohexene (**A**) undergoes an isomerization process to produce another carboncationic intermediate of methylcyclopentene (**B**). **B** is further stabilized to form a tertiary carbenium (**C**) or a secondary carbenium (**D**). **C** finally forms 1-methylcyclopentene through dehydrogenation and **D** forms 3-methylcyclopentene. Besides, a further charge transfer of **D** forms another secondary carbenium **E** and 4-methylcyclopentene can be generated subsequently. Besides, **A** and **B** could also form byproducts cyclohexane (cha) and methylcyclopentane (mcpa) through hydrogen transfer, respectively, which are also detectable with limited selectivity in the experiments (Table S10). In general, tertiary carbenium is more stable than secondary carbenium because the positive charge can be better dispersed through three groups linked to the carboncationic center. Therefore, 1-methylcyclopentene should be the main product. Comparing two secondary carbeniums (**D** and **E**), **E** can only be formed through an extra charge transfer process, and thus the formation probability of 4-methylcyclopentene should be lower than that of 3-methylcyclopentene. In summary, the relative content of three different isomers should follow the sequence of 1-methylcyclopentene > 3-methylcyclopentene > 4-methylcyclopentene.

**Fig. 6:**
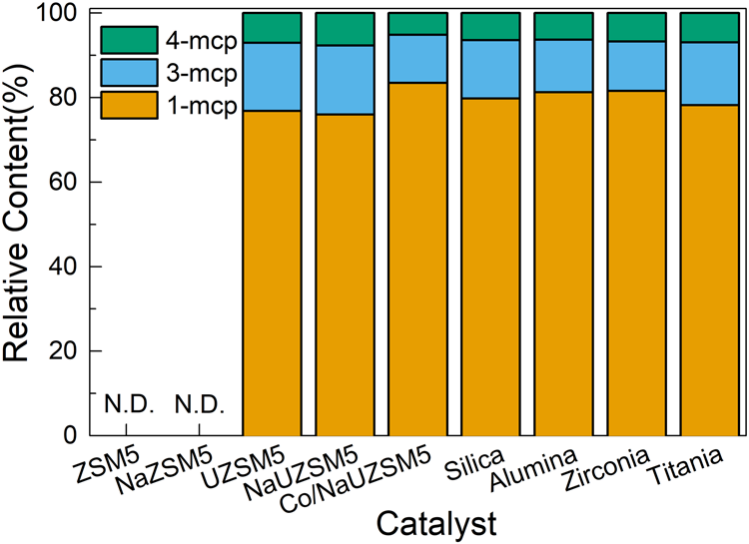
Relative content of three methylcyclopentene isomers formed over various catalysts. N.D. indicates no methylcyclopentene product was detected.

In order to confirm the proposed mechanism, the relative content of three methylcyclopentene isomers collected from all runs are compared in Fig. 6. As expected, the relative content follows the same sequence in all entries (within 10% relative error): 1-methylcyclopentene > 3- methylcyclopentene > 4- methylcyclopentene, thus supporting the proposed carboncationic mechanism. This sequence is also consistent with previous literature report^7^. Besides, a further thermodynamic equilibrium calculation also coincides well with experimental results (Table S12), further confirming the reliability of the conclusion. It is suggested that despite the intrinsic differences of charged catalytic materials, the cyclohexene skeletal isomerization process can take place by following a similar mechanism.

### Effect of Co addition

Based on the aforementioned mechanism and characterization results, the effects of Co introduction can also be interpreted. NH_3_-TPD results in Table S4 indicate that the presence of Co leads to the transition of weak acid sites to strong acid sites, which can also be clearly seen by the emergence of a new peak at 370 °C in Fig. S6. Moreover, the peaks of Lewis acid sites are also intensified in DRIFTS analyses, as shown in Fig. S7. These observations suggest that Co interacts with NaUZSM-5 catalyst to generate more strong Lewis acid sites, facilitating the adsorption of cyclohexene. This effect results in slightly higher cyclohexene conversion, and the methylcyclopentene selectivity can also be improved because of well-controlled Bronsted acidity.

**Fig. 7:**
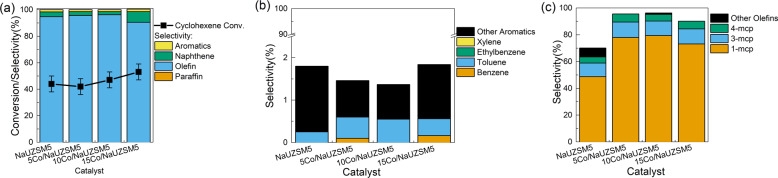
Performances of NaUZSM-5 with different Co loading for catalytic cyclohexene conversion. **a** Cyclohexene conversion and PONA selectivity. **b** Selectivity of aromatic species. **c** Selectivity of olefin species. The error bars represent the standard deviation of cyclohexene conversion measurement.

In order to get more clues of the Co effect, a series of NaUZSM-5 catalysts with different Co contents were prepared and evaluated, and the results are shown in Fig. 7. According to Fig. 7a, cyclohexene conversion increases gradually along with increasing Co contents, which can be due to stronger cyclohexene adsorption. As expected, better olefin selectivity can also be observed when Co content increases from 0 to 10 wt.%. However, lowered olefin selectivity is observed when Co content reaches 15 wt.%, which might be due to the blocking of pore structures caused by excessive Co loading. This hypothesis is further supported by N_2_ adsorption/desorption analysis of Co/NaUZSM-5 catalysts with different Co loadings (Table S7): the micropore surface area and pore volume decline significantly along with increasing Co content. The consistency between experimental results and anticipations further confirms the conclusions regarding the interaction between Co and zeolite catalyst.

In order to check the specificity of Co addition, several other metal components including Mo, Ag, Ga and Ce were loaded on NaUZSM-5 and the catalytic performances were evaluated as illustrated in Fig. S11. It is observed that Co leads to the highest olefin selectivity, while the other metals result in lower olefin selectivity compared to NaUZSM-5. More specifically, Mo leads to high aromatic selectivity and Ag, Ga, Ce cause the enhancement of selectivity towards paraffins and naphthenes, which are not desirable for methylcyclopentene production. These catalysts were further subject to NH_3_-TPD analysis to determine the properties of acid sites, and corresponding results are shown in Fig. S12 and Table S8. It should be noted that the peak of strong acid sites at 370 °C is unique after the introduction of Co, which is not found in catalysts with other metal components. Mo leads to significantly enhanced acidity, which explains the high aromatic selectivity. The presence of Ag, Ga, and Ce slightly changes the amount and NH_3_ desorption patterns of weak acid sites, resulting in the variation of product selectivity. According to the results, it is further substantiated that Co finely modulates the distribution of weak and strong acid sites by interacting with zeolite support and thus improves methylcyclopentene selectivity, which is distinctive among all tested metals.

### Location preference: external surface vs. internal pores of zeolite structure

**Fig. 8:**
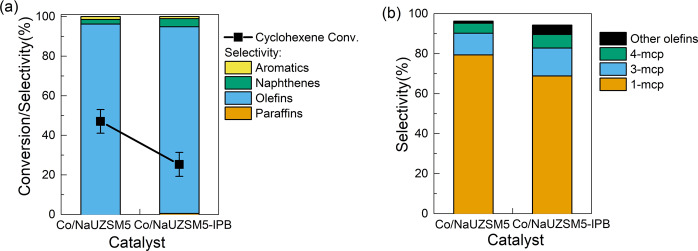
Performances of Co/NaUZSM5 and Co/NaUZSM5-IPB for catalytic cyclohexene conversion. **a** Cyclohexene conversion and PONA selectivity. **b** Selectivity of olefin species. The error bars represent the standard deviation of cyclohexene conversion measurement.

To further investigate the location preference of cyclohexene skeletal isomerization over zeolite-based catalyst, Co/NaUZSM-5 catalyst was treated with an innovative inner pore blocking technique and the produced catalyst was labeled as Co/NaUZSM-5-IPB. N_2_ adsorption/desorption isotherms of Co/NaUZSM-5 and Co/NaUZSM-5-IPB catalysts are compared in Fig. S13 and the derived structural properties are listed in Table S9. According to Table S9, the micropore surface area of Co/NaUZSM-5-IPB is lower than Co/NaUZSM-5, while the external surface area remains almost unchanged, indicating the inner pore structure of Co/NaUZSM-5 is successfully and exclusively blocked to a certain degree. Then, the catalytic performances of two catalysts are compared in Fig. 8. From Fig. 8a, an apparent decrease of cyclohexene conversion from 47% to 25% is observed, accompanied with the lowered olefin selectivity. Detailed analysis of olefin species in Fig. 8b also demonstrates that the selectivity towards methylcyclopentene, particularly 1-methylcyclopentene, is also declined. Since the external surface area of two catalysts is similar, the deteriorated catalytic performance of Co/NaUZSM-5-IPB could only be attributed to the partially blocked inner pore structure. Therefore, the cyclohexene skeletal reaction is experimentally proved to preferably proceed in the inner pores of zeolite-based catalysts.

**Fig. 9:**
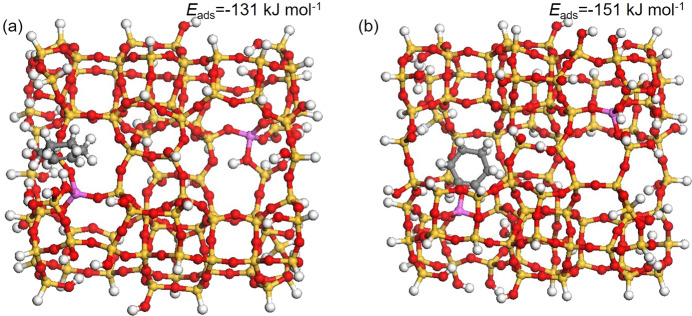
Optimized structure of adsorbed cyclohexene. **a** On the external surface. **b** Within the internal pore of zeolite-based catalyst. The corresponding adsorption energies *E*_ads_ are also given. Colors of atoms: yellow for Si, magenta for Al, red for O, white for H and gray for C.

In order to strengthen this conclusion, corresponding DFT calculation was also carried out. The optimized structures of cyclohexene adsorption on the external surface and within the internal pore of zeolite-based catalyst are shown in Fig. 9. According to the calculation results, the adsorption energy of cyclohexene in the internal pore of zeolite-based catalyst is 20 kJ mol^−1^ lower than that on the external surface, strongly supporting the reaction prefers to take place in the internal pores rather than on the external surface of the catalyst structure.

### Different acidity dependence: zeolite-based catalyst vs. metal-oxide-based catalysts

As mentioned before, a negative correlation between methylcyclopentene selectivity and the number of acid sites in zeolite-based catalysts is observed, which is completely contrary to that over conventional metal-oxide-based catalysts. Based on the discussion above, a more in-depth explanation can be proposed regarding the different dependence on the number of acid sites over these two types of catalysts. According to N_2_ adsorption/desorption analysis, the pore size of zeolite-based catalysts is proved to be within the range of 0.55–0.6 nm, which matches well with the kinetic diameter of cyclohexene^26^. Therefore, the cyclohexene molecules diffused within the inner pore will have strong space confinement and get full contact with the internal wall of the zeolite structure. Under these circumstances, if there are abundant acidic sites, it is highly possible that cyclohexene will interact with more than one acidic site, resulting in uncontrollable over-protonation and the well-recognized aromatization pathway for benzene formation^5^. Therefore, only limited and sparsely distributed acidic sites on the surface of zeolite-based catalysts will favor the isomerization process. On the other side, metal-oxide-based catalysts engaged in this study are all mesoporous materials with much larger pore size. Therefore, there is almost no space confinement for cyclohexene adsorption, resulting in single-site adsorption regardless of the number of acid sites. Then, the presence of more acidic sites leads to the adsorption of more cyclohexene molecules and higher isomerization activity can thus be expected. In summary, the opposite dependence on the number of acid sites over zeolite-based and metal-oxide-based catalyst might be mainly due to the different space confinement of cyclohexene adsorption caused by pore size distribution of the charged catalytic material, implying that the reaction might be highly shape selective.

## Conclusions

In conclusion, highly selective skeletal isomerization of cyclohexene to produce methylcyclopentene with high purity is realized over zeolite-based catalysts. K plays an important role during the synthesis of UZSM-5, which noticeably changes the number of acid sites and the pore structure of zeolite. Co can interact with UZSM-5 by modifying the type and strength of acid sites, which finely tunes the catalytic performances. 96.8 wt.% high liquid yield and 95.8 wt.% methylcyclopentene selectivity can be achieved over Co/NaUZSM-5 with satisfactory stability. A similar carboncationic mechanism of skeletal isomerization is observed overall catalysts under current investigation, leading to a consistent distribution of three methylcyclopentene isomers. The skeletal isomerization reaction is proved to take place more preferably in the inner pores rather than on the external surface of zeolite structure through employing an innovatively selective inner pore blocking strategy. DFT study was further performed and the simulation results are in great agreement with experimental observations. Finally, the reverse dependence on the number of acid sites over zeolite-based and metal-oxide-based catalyst suggests the pore structure of catalytic material is also essential for catalytic isomerization performances due to space confinement effects. This work provides extra insight into catalytic cyclohexene conversion in terms of selective formation of desired high-value products methylcyclopentene. Although zeolite materials are well recognized for catalyzing aromatization reactions, it is found that they can also be used for typical isomerization reactions if the acidity is effectively controlled. The knowledge gained from this study thus provides new strategies for preparing catalysts with maximized isomerization and limited aromatization selectivity, which can also be used as a platform material for other reaction systems where isomerization is preferred.

## Methods

### Material and catalyst syntheses

The traditional ammonium-type ZSM-5 zeolites with the silica to alumina ratio of 80:1 was purchase from Zeolyst USA. The NH_4_-type zeolite supports in powder form were converted to H-type by calcination in static air at 600 °C for 3 h. The obtained ZSM-5 was stored in sealed vials for further use. The uniform compact cylindrical shape zeolite (UZSM-5) with the same silica to alumina ratio of 80:1 was synthesized according to our previous work^27^. Typically, 5.4020 g aluminum nitrate (Al(NO_3_)_3_·9H_2_O, 98%, Alfa Aesar) as the Al precursor was added into 100 mL 0.1 mol L^−1^ template solution tetrapropylammonium hydroxide (TPAOH, Sigma Aldrich). Then, 120 g tetraethyl orthosilicate (TEOS, 98%, Sigma Aldrich) as the silica source was firstly added dropwise through a burette into the aforementioned solution. The solution was further stirred for 2 h and put in a 500 mL Teflon lined autoclave. Next, the autoclave was sealed, heated up to 170 °C, and held for 72 h for hydrothermal synthesis. After the hydrothermal treatment, the product was collected and rinsed three times by deionized water through centrifugation. Finally, the catalyst structure was obtained through calcination at 600 °C in static air for 3 h so that the template agent was removed. The final UZSM-5 catalyst structure was stored in sealed vials for further use. It should be noted that a considerable amount of potassium is present in UZSM-5, which can be attributed to the high K content in template solution TPAOH. According to the calculation, the K contained in TPAOH (0.013 mol) is sufficient to provide all detected K in UZSM-5 (0.011 mol) in a typical synthesis process. NaZSM-5 and NaUZSM-5 catalyst was prepared from ZSM-5 and UZSM-5 catalyst, respectively through an ion exchange process. In a typical ion exchange cycle, 1 g zeolite was put in 50 mL 0.01 mol L^−1^ sodium hydroxide (NaOH, 98%, Sigma Aldrich) aqueous solution and set for 5 minutes at room temperature, followed by a centrifugation process to separate the catalyst and the solution. NaOH was selected as the Na^+^ source to prevent the introduction of other undesired anions (e.g. Cl^−^), and the temperature and contact time of the ion exchange process were controlled to avoid the destruction of the zeolitic structure due to its solubility in basic condition. The ion exchange cycle was repeated three times, and the final product was fully rinsed by deionized water and calcined at 600 °C in static air for 3 h. The obtained NaZSM-5 and NaUZSM-5 catalyst structures were stored in sealed vials for further use. Co/NaUZSM-5 with 10 wt.% cobalt was prepared from NaUZSM-5 through impregnation using 1 mol L^−1^ aqueous solution of Co(NO_3_)_2_·6H_2_O (98%~102%, Alfa Aesar) and further calcination at 550 °C for 2 h. Besides, silica (SiO_2_, Alfa Aesar), gamma-alumina (γ-Al_2_O_3_, 20 nm, 99%, Alfa Aesar), zirconia (ZrO_2_, Alfa Aesar), and titania (TiO_2_, 99.5%, 21 nm, Sigma-Aldrich) supports were purchased and fully dried at 100 °C overnight before usage.

Besides, selective internal-pore blocked Co/NaUZSM-5 catalyst was prepared using a similar method reported in our previous work^20^. First, 1 g Co/NaUZSM-5 was ion exchanged by 50 mL 0.1 mol L^−1^ aqueous solution of Ca(NO_3_)_2_·4H_2_O (99%, Alfa Aesar) at 80 °C for three times, followed by a calcination process at 600 °C in static air for 2 h. Then, the obtained catalyst was put in a tube furnace and heated up to 600 °C under 100 sccm (standard cubic centimeter per minute) N_2_ flow for 2 h. Next, 100 sccm ethylene was introduced to selectively block the internal pores of zeolite catalysts by the coking process for 1 h. After cooling down to room temperature, the catalyst was further ion exchanged by 0.01 mol L^−1^ HNO_3_ to eliminate the remaining Ca species. Finally, the catalyst was heated up again in a tube furnace at 600 °C under 100 sccm N_2_ flow for 2 h. The acquired catalyst was named Co/NaUZSM-5-IPB and stored in sealed vials for further use.

### Reaction process

The catalytic cyclohexene conversion was carried out under unreactive nitrogen atmosphere in a batch reactor at 400 °C and atmospheric pressure for 1 h. A typical reaction process was as follows. First, 0.2000 g catalyst and 2.00 g cyclohexene (99%, Alfa Aesar) were put in a 100 mL batch reactor procured from Parr Instrument USA with the maximum temperature and pressure tolerance at 500 °C and 5000 psi (34.5 MPa), respectively. Then, the reactor was sealed by flange with a reactor cap, which was designed to measure the temperature by thermocouple and the pressure by pressure gauge. The reactor was also able to facilitate the mass transfer in the reactor by a magnetic stirrer and accurately control the reaction condition by a cooling water system. Next, a N_2_ standard gas (99.999%, Air Liquide) was used to purge the reactor three times through a connected tubing system so that the other components in air could be fully removed from the reactor. Subsequently, the reactor was set into a heating mantle and heated up to the desired reaction temperature 400 °C through proportional-integral-derivative (PID) controlling system after passing a leak test. The heating up stage took about 20 minutes and the reaction lasted another 1 h after the temperature reached the set point. The reaction process was recorded by videos and a typical temperature curve is shown in Fig. S1. When the scheduled reaction time was met, the reactor was cooled down by blowing air flow at room temperature.

### Product analyses

After the reaction, the products in gas, liquid and solid phase were collected respectively and analyses were carried out in following manners.

For the gas phase, the mass of gas after the reaction was determined by the mass difference of reactor before and after the degassing process. Then, the product gas was directly fed into a micro-chromatography (micro-GC) to get the gas composition. Instrument and conditions: four-channel micro-GC (490, Agilent) equipped with thermal conductivity detector (TCD). The first channel was equipped with 10 m molecular sieve 5 A column to analyze H_2_, O_2_, N_2_, CH_4,_ and CO; the second channel was equipped with 10 m PPU column to analyze CO_2_, C_2_H_6_, and C_2_H_4_; the third channel was equipped with 10 m alumina column to analyze C_4_~C_6_ hydrocarbons; and the forth channel was equipped with 8 m CP-Sil 5CB column to analyze propane and propylene. Temperature and pressure of columns: 80 °C and 200.0 kPa (first), 100 °C and 175.0 kPa (second), 80 °C and 180.0 kPa (third), 100 °C and 85.0 kPa (fourth), analysis time: 2.5 min. The carrier gas was Ar in all columns. The composition of gas products is shown in Table S1.

For the liquid phase, the degassed product was first filtrated through a medium rate qualitative filter paper to separate the liquid product and the remaining solid. The collected liquid products were further dissolved in carbon disulfide (CS_2_, 99%, Sigma Aldrich) and injected into a gas chromatography-mass spectroscopy (GC-MS) to get the corresponding composition. In order to obtain a better understanding of the liquid product composition, the designated hydrocarbon species were classified into four main groups according to their structural characteristics. The four classifications were represented by PONA, namely paraffin (alkanes), olefin (alkenes and cycloalkenes), naphthene (cycloalkanes), and aromatics (compounds with one or more benzene ring). Instrument and conditions: GC-MS (PerkinElmer GC Claus 680 and MS Clarus SQ 8 T) equipped with a capillary column which was designed to analyze paraffin-olefin-naphthene-aromatic (PONA) components (Agilent HP-PONA). A stepwise temperature ramping program was set during the analysis and the corresponding curve was shown in Fig. S2. Standard PONA species with 5–11 carbon numbers were used to calibrate the GC-MS by establishing a working curve to correlate the concentration of each species and the peak area in chromatogram, so that the amount of each product in the liquid phase can be determined. During the analysis process, the peaks in chromatogram were designated by comparing the mass spectra with standard compounds in National Institute of Standards and Technology (NIST) database and selecting the most probable hit. The unreacted cyclohexene was quantified and excluded when product analysis was performed.

Besides, the collected solid was well mixed and a certain amount of sample was put in an alumina boat for thermogravimetric analysis (TGA) to determine the amount of coke formation during the process. TGA measurement was performed with a simultaneous thermal analyzer (PerkinElmer STA 6000). The samples were held at 30 °C for 5 min for a stable initial weight, then ramped to 800 °C at a rate of 20 °C min^−1^ under 30 mL min^−1^ air flow and held for 5 min. The weight loss from 350 °C to 800 °C was attributed to the oxidation of coke and the overall coke yield was calculated by assuming the coke was evenly distributed on the used catalyst structure. The corresponding TGA curves were shown in Fig. S3.

After collecting all relevant data, the corresponding values were calculated according to the following equations. The overall performances with different catalysts are listed in Table S2.

$${\mathrm{Overall}}\,{\mathrm{mass}}\,{\mathrm{balance}}\,{\mathrm{=}}\frac{{{\mathrm{mass}}\,{\mathrm{of}}\,\left( {{\mathrm{gas}} + {\mathrm{liquid}} + {\mathrm{solid}}} \right)\,{\mathrm{after}}\,{\mathrm{reaction}}}}{{{\mathrm{mass}}\,{\mathrm{of}}\,\left( {{\mathrm{gas + liquid + solid}}} \right)\,{\mathrm{before}}\,{\mathrm{reaction}}}} \times 100\%$$

$${\mathrm{Gas}}\,{\mathrm{yield}} = \frac{{{\mathrm{mass}}\,{\mathrm{of}}\,{\mathrm{produced}}\,{\mathrm{gas}}\,{\mathrm{in}}\,{\mathrm{product}}}}{{{\mathrm{mass}}\,{\mathrm{of}}\,{\mathrm{fed}}\,{\mathrm{cyclohexene}}}} \times 100\%$$

$${\mathrm{Methane}}\,{\mathrm{Conversion}} = \left( 1-{\frac{{{\mathrm{mole}}\,{\mathrm{of}}\,{\mathrm{remaining}}\,{\mathrm{methane}}\,{\mathrm{after}}\,{\mathrm{reaction}}}}{{{\mathrm{mole}}\,{\mathrm{of}}\,{\mathrm{fed}}\,{\mathrm{methane}}}}} \right) \times 100\%$$

$${\mathrm{Liquid}}\,{\mathrm{yield}} = \frac{{{\mathrm{mass}}\,{\mathrm{of}}\,{\mathrm{liquid}}\,{\mathrm{in}}\,{\mathrm{product}}}}{{{\mathrm{mass}}\,{\mathrm{of}}\,{\mathrm{fed}}\,{\mathrm{cyclohexene}}}} \times 100\%$$

$${\mathrm{Coke}}\,{\mathrm{yield}} = \frac{{{\mathrm{mass}}\,{\mathrm{of}}\,{\mathrm{coke}}\,{\mathrm{in}}\,{\mathrm{product}}}}{{{\mathrm{mass}}\,{\mathrm{of}}\,{\mathrm{fed}}\,{\mathrm{cyclohexene}}}} \times 100\%$$

$${\mathrm{Selectivity}}\,{\mathrm{of}}\,{\mathrm{S}} = \frac{{{\mathrm{mas}}\,{\mathrm{of}}\,{\mathrm{S}}\,{\mathrm{in}}\,{\mathrm{product}}}}{{{\mathrm{mass}}\,{\mathrm{of}}\,{\mathrm{converted}}\,{\mathrm{cylohexene}}}} \times 100\%$$

Here, S can represent a specific species or the collection of a species group.

### Catalyst characterizations

N_2_ adsorption-desorption analysis was carried out on ASAP 2020 Plus surface area and porosimeter system (Micromeritics) to get the structural properties of catalysts. In a typical run, the sample was first degassed at 350 °C for 4 h with a temperature ramping rate of 10 °C min^−1^ and a vacuum level of 20 μmHg. Then the analysis was performed in liquid nitrogen to get a 56-point adsorption-desorption isotherm. The total surface area was calculated by BET method and the total pore volume was calculated at 0.995 relative pressure. Micropore surface area and volume were obtained by the t-plot method, and average pore size was acquired using the BJH method. Besides, micropore size distribution was calculated by Horvath-Kawazoe method and the overall pore size distribution was based on BJH method. The adsorption/desorption isotherm is shown in Fig. S4. Surface area and pore volume analysis results are listed in Table S3 and pore size distribution is further illustrated in Fig. S5.

Ammonia-temperature programmed desorption (NH_3_-TPD) was performed to determine the surface acidity of zeolite catalysts on a chemisorption analyzer (Finesorb-3010). Typically, 0.2 g catalyst was put into a U-type quartz tube and both ends were filled with quartz wool. In order to remove the adsorbed impurities, a temperature programmed oxidation (TPO) test was first performed, in which the tube was heated up to 600 °C and held for 30 minutes with a ramp rate of 20 °C min^−1^ under 5%O_2_/He gas flow (flow rate 30 sccm). Then, the system was cooled down to 120 °C and ammonia adsorption was conducted by feeding 10% NH_3_/He for 30 minutes (flow rate 25 sccm). Next, the physisorbed ammonia was flushed out by He gas flow for 30 minutes (flow rate 30 sccm). Finally, the desorption of ammonia was carried out from 120 °C to 800 °C with a ramping rate of 20 °C min^−1^ and held at 800 °C for 10 minutes. The desorbed ammonia was monitored by a thermal conductivity detector (TCD) and the amount was quantified by peak integration of the corresponding calibrated TCD signal. The profiles are shown in Fig. S6 and the number of acid sites after quantification are tabulated in Table S4.

Diffuse reflectance infrared Fourier transform spectrometry (DRIFTS) was used to provide further information of the types of surface acidity by in-situ pyridine adsorption analysis. Instrument and conditions: FT-IR spectrometer (Thermo-Scientific-Nicolet iS50) equipped with an mercury-cadmium- telluride (MCT) detector. The sample was first treated at 500 °C with 30 sccm N_2_ flow for 15 min to clean up the adsorbed impurities. Then, the temperature was reduced to 25 °C with continuous N_2_ flow until the background test showed no characteristic peak of CO_2_. Next, pyridine was carried by N_2_ through a bubbler and introduced into the sampling chamber for 15 minutes. Finally, pure N_2_ gas was used again to purge out unadsorbed pyridine for another 15 minutes, and the FT-IR spectra were obtained based on the background in the previous step. Measurement parameters: number of scans: 512, detection mode: adsorption, data spacing: 0.241 cm^−1^. The spectra with peak designations are shown in Fig. S7.

Inductively coupled plasma-optical emission spectrometry (ICP-OES) was used to determine the content of important elements in catalyst samples. 50 mg of catalyst sample was put in a Teflon liner. Then, 6 mL 68 wt.% HNO_3_ and 2 mL 40 wt.% HF was added and the sample was sealed and digested in a microwave digestor (Anton Paar, Multiwave 5000). The obtained liquid was further diluted by 2 wt.% HCl as the matrix for ICP-OES measurement (Thermo Scientific, iCAP 7000 ICP spectrometer). The results are tabulated in Table S5.

Scanning electron microscope (SEM) was used to investigate the morphology of zeolite catalysts. Instrument and conditions: FEI Quanta 250 FEG variable-pressure/environmental field emission scanning electron microscope, operation voltage: 5 kV. The images are presented in Fig. S8.

X-ray diffraction (XRD) was used to confirm the crystal structure of zeolite catalysts. Instrument and conditions: Rigaku multiflex diffractometer with Cu Kα irradiation, working voltage: 20 kV, working current: 40 mA. Scanning range: 2θ = 3°–60°, scanning speed: 3° min^−1^. The corresponding patterns are shown in Fig. S8.

### DFT calculations

The density functional theory (DFT) calculation was carried out using the DMol3 module in Materials Studio software (Accelrys Inc.). B3LYP hybrid functional was used for the calculation of exchange-correlation. The double numeric quality basis set with the gradient corrected GGA was adopted, which has one set of occupied atomic orbital and a second set of valence atomic orbital^28,29^. A polarization d-function on all non-hydrogen atoms and a polarization p-function on all hydrogen atoms were applied. This basis set could provide reasonable accuracy with acceptable computational cost, which was widely used in DFT calculation for zeolite-based catalysts. The tolerances of energy, gradient, and displacement convergence were 2 × 10^−5^ Ha, 4 × 10^−3^ Ha/Å, and 5 × 10^−3^ Å, respectively.

The initial HZSM-5 structure with a unit cell of Si_96_O_192_ was obtained from the database of International Zeolite Association Structure Commission (IZA-SC)^30^. All terminal Si and O atoms were saturated by H atoms. For all the geometry optimization processes, the bond length of terminal O–H and Si–H groups were fixed at 0.975 Å and 1.500 Å, respectively.

In order to satisfy the silica to alumina ratio of the catalyst, two out of 96 tetrahedral Si sites (T-sites) were replaced by Al atoms at T-12 sites due to the highest probability for replacement^31^. To balance the negative charge caused by Al replacement, an extra proton was introduced to form Al-O^H^-Si connection, forming Bronsted acid sites in the zeolite structure. The structure of zeolite as well as the free molecules were first optimized, followed by an adsorption energy calculation in which cyclohexene molecules were located directly next to the Bronsted acid sites as the initial inputs for optimization. The adsorption energy was calculated by the following equation, where *E*_ads_ is the adsorption energy, *E*_A-C_ is the energy of the adsorbed species together with the catalyst, *E*_A_ is the energy of the free adsorbate and *E*_C_ is the energy of the catalyst in its original form.

$$E_{{\mathrm{ads}}} = E_{{\mathrm{A - C}}} - E_{\mathrm{A}} - E_{\mathrm{C}}$$

### Thermal equilibrium calculations

Consider the equilibrium among three isomers of methylcyclopentene (mcp). The Gibbs free energy Δ*G* was obtained through DFT calculation and the equilibrium constant *K* was calculated by the following equation, where *T* is the reaction temperature (673.15 K) and *R* is the universal gas constant. The results are shown in Table S11.

$$\Delta G = - RT\ln K$$

According to the definition of equilibrium constant *K*, the concentration relationship between three methylcyclopentene isomers can be obtained. Then, the theoretical relative content of three isomers can be calculated. It is further compared with the average relative content in all experimental runs in Table S12.

## Supplementary information

Supplementary Information

## Data Availability

The datasets generated during and/or analyzed during the current study are available in the Figshare repository, 10.6084/m9.figshare.13726408 and 10.6084/m9.figshare.13726414.
