# Supplementary material for: Highly selective skeletal isomerization of cyclohexene over zeolite-based catalysts for high-purity methylcyclopentene production
Source: Commun Chem. 2021 Mar 11;4:34. doi: 10.1038/s42004-021-00472-8 (PMC9814423; doi:10.1038/s42004-021-00472-8)
Supplement: Supplementary file 1 — Supplementary Information [file 42004_2021_472_MOESM1_ESM.pdf]

Highly selective skeletal isomerization of cyclohexene over zeolite-based catalysts for high-purity methylcyclopentene production

Hao Xu, Zhaofei Li, Shijun Meng, Jack Jarvis, Hua Song*

Department of Chemical and Petroleum Engineering, University of Calgary, 2500 University Drive, NW, Calgary, Alberta T2N 1N4, Canada

*Corresponding author. E-mail: sonh@ucalgary.ca (H. Song).

Supplementary Information

Contents

Figures... 3

Fig. S1 Typical temperature curve during reaction process. 3

Fig. S2 Programmed oven temperature for GC-MS analysis. 4

Fig. S3 Thermalgravimetric analysis (TGA) and derivatives (DTG) of used catalysts............................. 5

Fig. S4 N_2 adsorption-desorption isotherms of different catalysts. 6

Fig. S5 Pore size distribution of catalysts. (a) Microporous zeolite-based catalysts, (b) Mesoporous metal oxide catalysts. 7

Fig. S6 NH_3-TPD profiles of zeolite catalysts. 8

Fig. S7 DRIFTS spectra of zeolite catalysts showing the characteristic pyridine adsorption on Bronsted acid sites (1485, 1492, 1586 and 1600 cm^{-1}) and Lewis acid sites (1445, 1485 and 1492 cm^{-1}). 9

Fig. S8 XRD patterns of ZSM-5 and UZSM-5 catalysts. 10

Fig. S9 SEM images of ZSM-5 and UZSM-5 catalysts. (a) ZSM-5, low magnification, (b) ZSM-5, high magnification, (c) UZSM-5, low magnification, (d) UZSM-5, high magnification. 11

Fig. S10 SEM images of potassium-free UZSM-5 catalysts. (a) low magnification, (b) high magnification. 12

Fig. S11 Performances of NaUZSM-5 loaded with various metals for catalytic cyclohexene conversion. 13

Fig. S12 NH_3-TPD profiles of NaUZSM-5 loaded with various metals. 14

Fig. S13 N_2 adsorption-desorption isotherms of Co/NaUZSM-5 and Co/NaUZSM-5-IPB catalysts. 15

Tables 16

Table S1 Gas product yields of catalytic cyclohexene conversion over different catalysts. 16

Table S2 Overall analysis results of catalytic cyclohexene pyrolysis over different catalysts. 17

Table S3 Structural properties of different catalysts. 18

Table S4 Surface acidity of zeolite-based and metal oxide-based catalysts derived from peak integration of NH_3-TPD signals. 19

Table S5 Content of several elements in zeolite-based catalysts determined by ICP-OES. 20

Table S6 Unit cell parameters for ZSM-5 and UZSM-5. 21

Table S7 Structural properties of UZSM-5 catalysts with different Co loadings. 22

Table S8 Surface acidity of NaUZSM-5 loaded with various metals derived from peak integration of NH_3-TPD signals. 23

Table S9 Structural properties of Co/NaUZSM-5 and Co/NaUZSM-5-IPB. 24

Tables S10 Detailed compositional analysis liquid products over zeolite-based and metal oxide-based catalysts for catalytic cyclohexene pyrolysis. The blank cells indicate the yield of corresponding product is below detection limit. 25

Table S11 Equilibrium calculation of methylcyclopentene isomers. 26

Table S12 Comparison of relative content among methylcyclopentene isomers derived from thermal equilibrium calculation and experimental result. 27

Figures

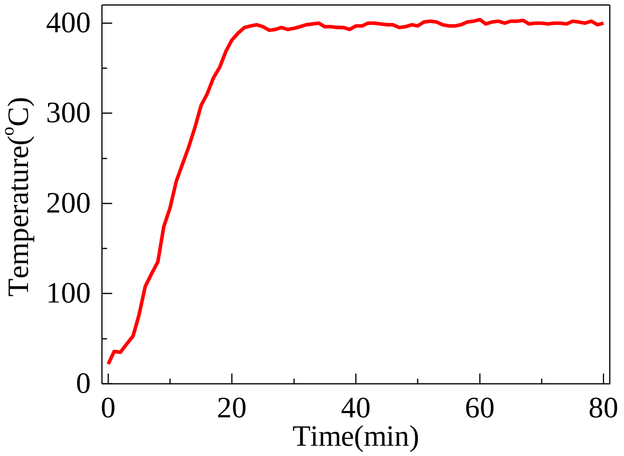

Fig. S1 Typical temperature curve during reaction process.

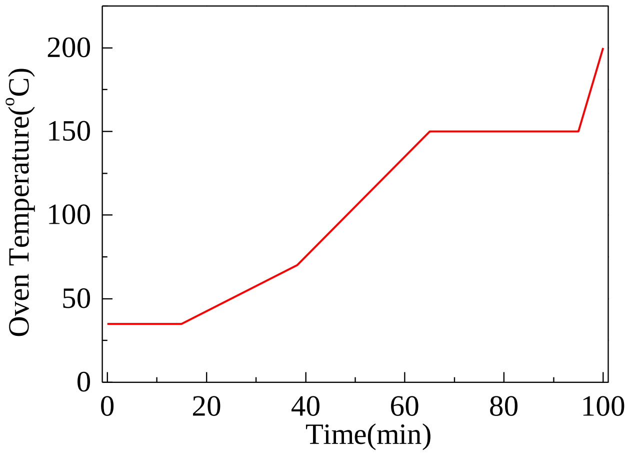

Fig. S2 Programmed oven temperature for GC-MS analysis.

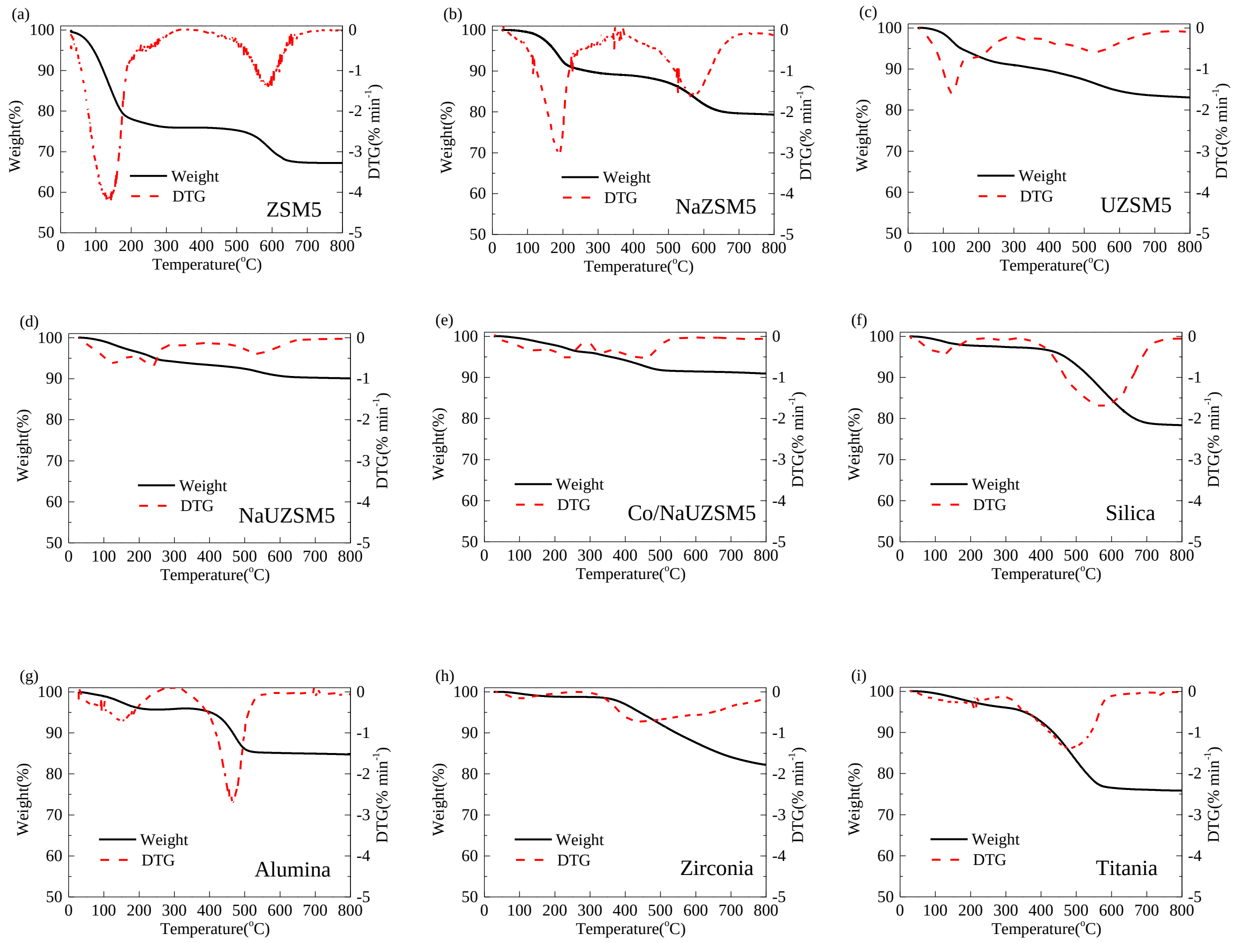

Fig. S3 Thermalgravimetric analysis (TGA) and derivatives (DTG) of used catalysts.

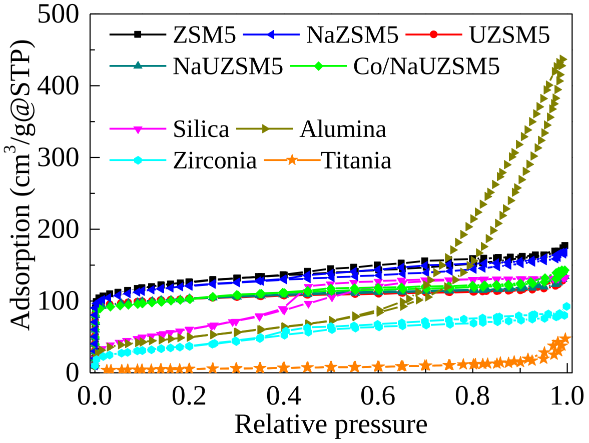

Fig. S4 N_2 adsorption-desorption isotherms of different catalysts.

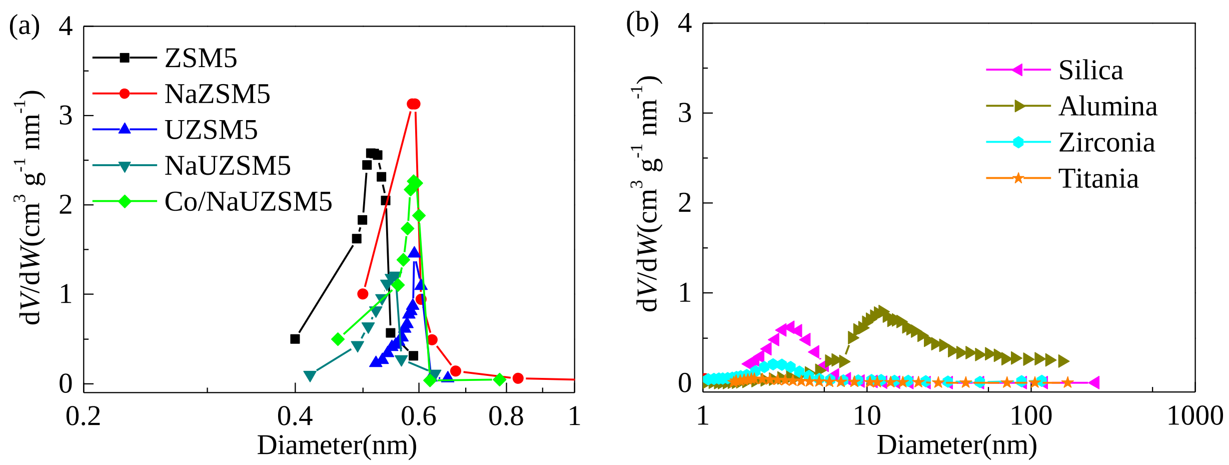

Fig. S5 Pore size distribution of catalysts. (a) Microporous zeolite-based catalysts, (b) Mesoporous metal oxide catalysts.

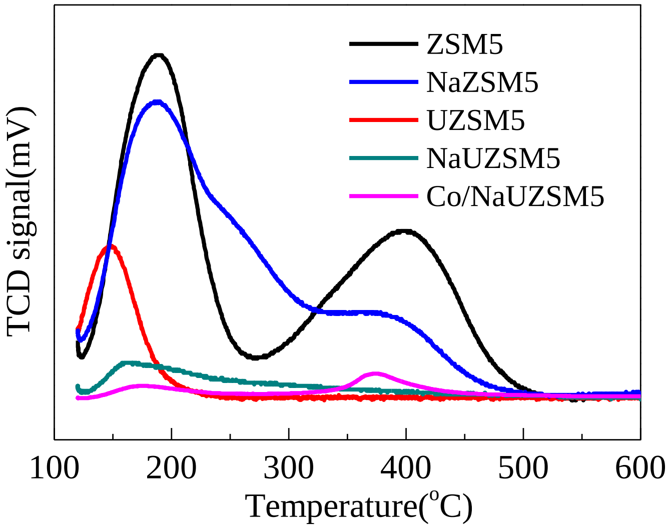

Fig. S6 NH_3-TPD profiles of zeolite catalysts.

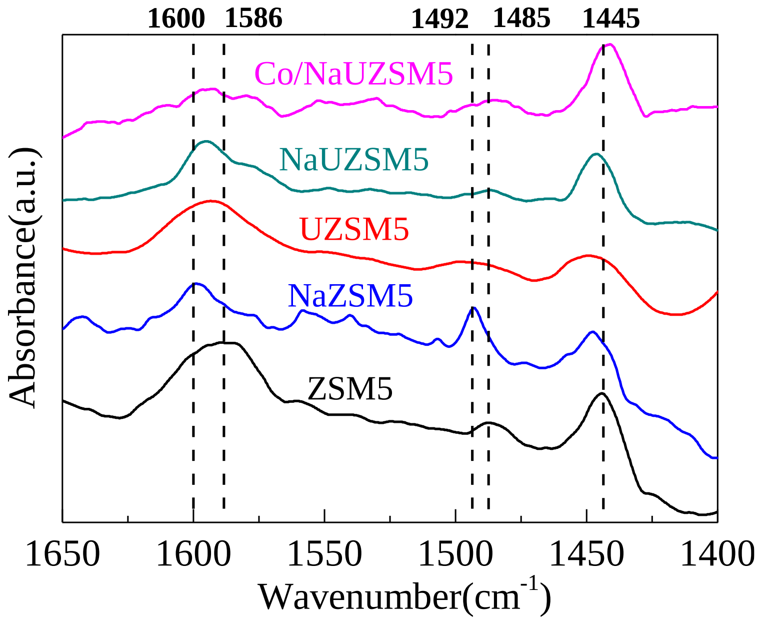

Fig. S7 DRIFTS spectra of zeolite catalysts showing the characteristic pyridine adsorption on Bronsted acid sites (1485, 1492, 1586 and 1600 cm^{-1}) and Lewis acid sites (1445, 1485 and 1492 cm^{-1}).

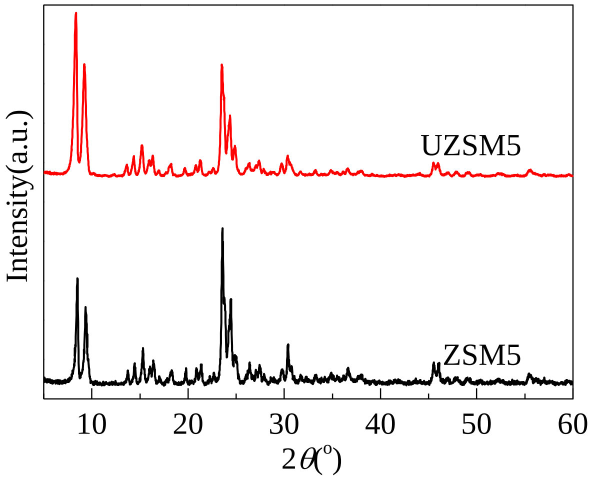

Fig. S8 XRD patterns of ZSM-5 and UZSM-5 catalysts.

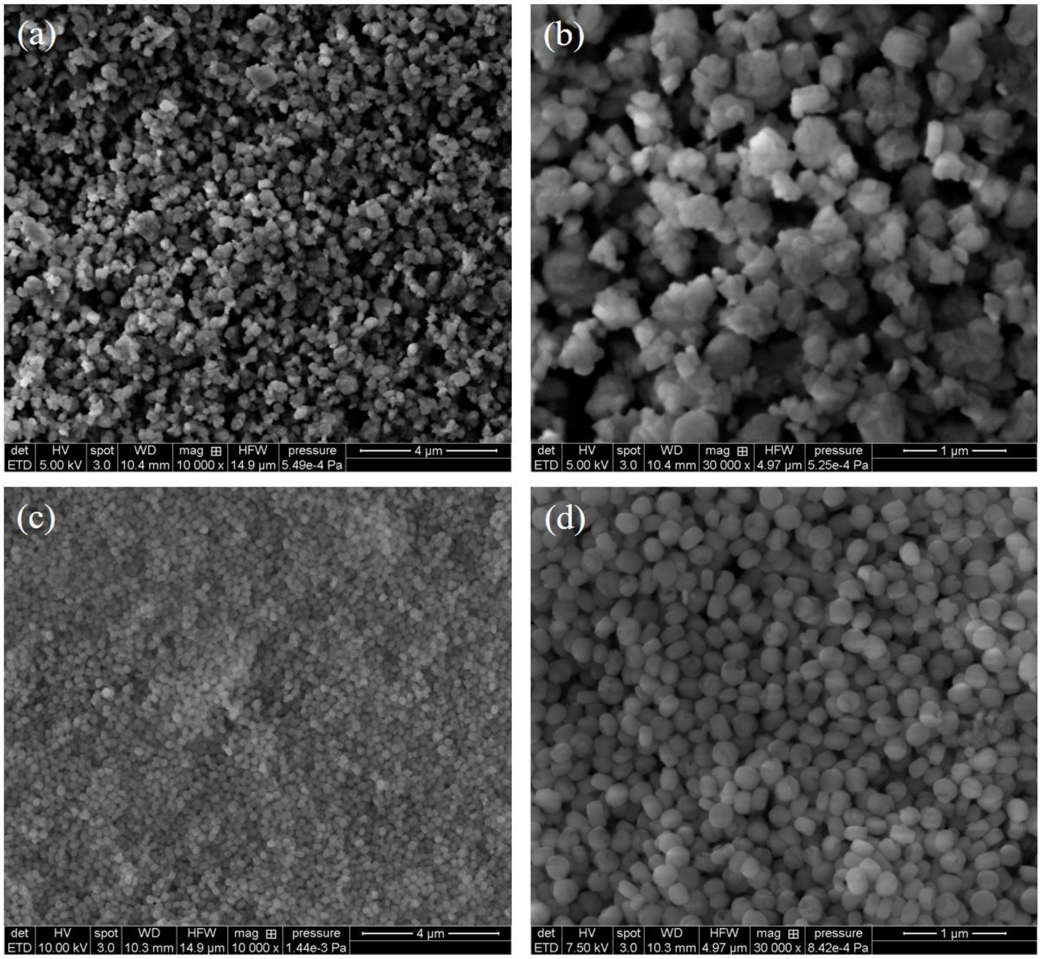

Fig. S9 SEM images of ZSM-5 and UZSM-5 catalysts. (a) ZSM-5, low magnification, (b) ZSM-5, high magnification, (c) UZSM-5, low magnification, (d) UZSM-5, high magnification.

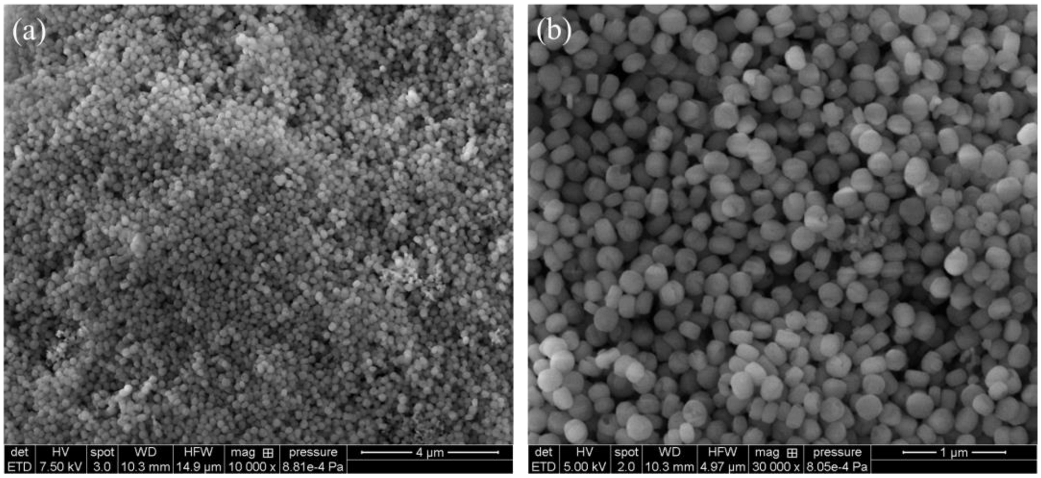

Fig. S10 SEM images of potassium-free UZSM-5 catalysts. (a) low magnification, (b) high magnification.

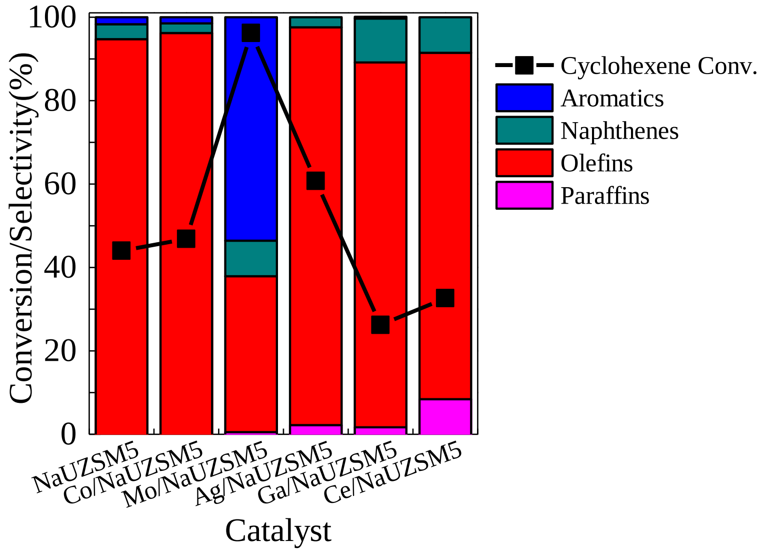

Fig. S11 Performances of NaUZSM-5 loaded with various metals for catalytic cyclohexene conversion.

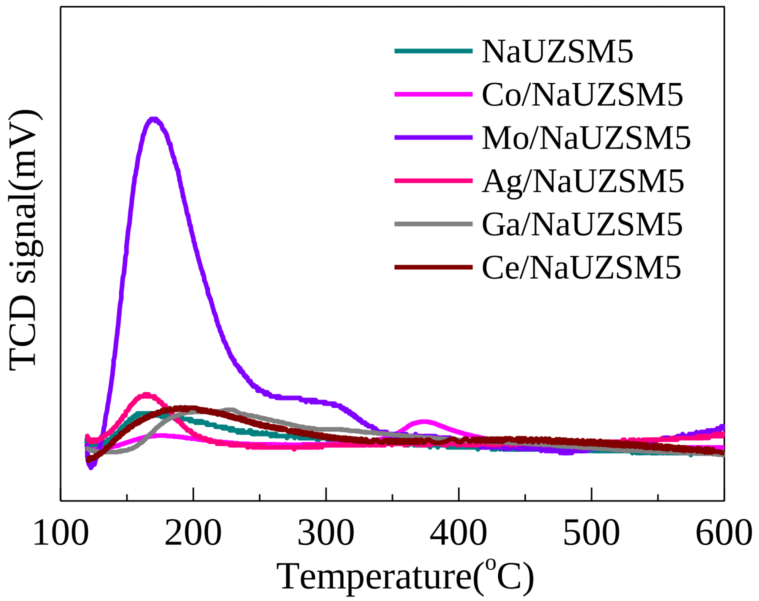

Fig. S12 NH_3-TPD profiles of NaUZSM-5 loaded with various metals.

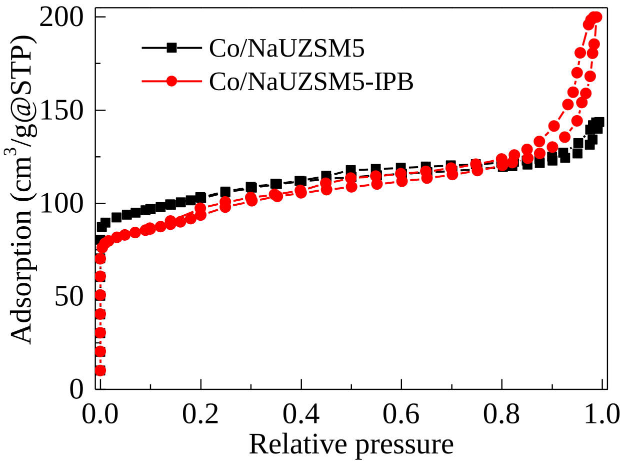

Fig. S13 N_2 adsorption-desorption isotherms of Co/NaUZSM-5 and Co/NaUZSM-5-IPB catalysts.

Tables

Table S1 Gas product yields of catalytic cyclohexene conversion over different catalysts.

Catalyst	H_2 yield (wt%)	CH_4 yield (wt%)	C2 yield (wt%)	C3 yield (wt%)	C4 yield (wt%)
None	0.02	BDL*	BDL	BDL	BDL
ZSM-5	0.21	0.03	3.55	5.58	2.72
NaZSM-5	0.34	0.02	5.17	3.52	0.55
UZSM-5	0.26	BDL	1.56	0.98	1.95
NaUZSM-5	0.23	BDL	1.10	1.69	0.24
Co/NaUZSM-5	0.18	BDL	0.26	0.11	0.15
Silica	0.14	BDL	1.66	1.31	0.29
Alumina	0.01	0.59	2.75	0.38	0.72
Zirconia	0.01	0.24	0.84	0.10	BDL
Titania	0.06	0.62	0.82	0.48	BDL

*BDL: below detection limit

Table S2 Overall analysis results of catalytic cyclohexene pyrolysis over different catalysts.

Catalyst	Cyclohexene conversion (%)	Gas yield (wt%)	Liquid yield (wt%)	Coke yield (wt%)	Overall mass balance (%)
None	7	0.5	98.5	0	99.0
ZSM-5	100	12.3	88.5	1.3	102.1
NaZSM-5	100	10.0	88.8	1.2	100.0
UZSM-5	93	6.0	91.2	0.9	98.1
NaUZSM-5	44	3.3	94.8	0.4	98.5
Co/NaUZSM-5	47	1.5	96.8	0.4	98.8
Silica	35	3.5	94.8	1.4	99.6
Alumina	41	7.0	92.6	1.3	100.9
Zirconia	23	4.0	95.0	1.2	100.2
Titania	45	5.0	92.5	2.7	100.2

Table S3 Structural properties of different catalysts.

Catalyst	BET surface area (m^2 g^{-1})	Total Pore volume (cm^3 g^{-1})	Micropore surface area (m^2 g^{-1})	t-plot Micropore volume (cm^3 g^{-1})
ZSM-5	395	0.27	222	0.12
NaZSM-5	377	0.25	221	0.12
UZSM-5	318	0.20	241	0.13
NaUZSM-5	319	0.20	235	0.12
Co/NaUZSM-5	327	0.22	192	0.10
Silica	229	0.21	0	0
Alumina	173	0.68	18	0.01
Zirconia	139	0.12	2	0
Titania	18	0.06	0	0

Table S4 Surface acidity of zeolite-based and metal oxide-based catalysts derived from peak integration of NH_3-TPD signals.

Catalyst	Weak site acidity (mmol g^{-1})	Strong site acidity (mmol g^{-1})	Total acidity (mmol g^{-1})
ZSM-5	232	184	417
NaZSM-5	293	22	315
UZSM-5	62	0	62
NaUZSM-5	32	0	32
Co/NaUZSM-5	8	16	24
Silica	15	0	15
Alumina	47	0	47
Zirconia	115	13	128
Titania	45	23	67

Table S5 Content of several elements in zeolite-based catalysts determined by ICP-OES.

Catalyst	Na (wt%)	K (wt%)	Co (wt%)
ZSM-5	BDL*	BDL	BDL
NaZSM-5	0.32	0.003	BDL
UZSM-5	BDL	1.23**	BDL
NaUZSM-5	1.07	0.99	BDL
Co/NaUZSM-5	0.99	1.01	9.04

*BDL: below detection limit

**The presence of K in UZSM-5 and relevant catalysts can be due to high potassium content (0.49 wt%) in template solution TPAOH

Table S6 Unit cell parameters for ZSM-5 and UZSM-5.

Sample	a (Å)	b (Å)	c (Å)	Volume (Å^3)
ZSM-5	19.7928	19.1059	12.8254	4850.04
UZSM-5	19.6728	19.3627	13.1278	5000.61

Table S7 Structural properties of UZSM-5 catalysts with different Co loadings.

Catalyst	BET surface area (m^2 g^{-1})	Total Pore volume (cm^3 g^{-1})	Micropore surface area (m^2 g^{-1})	t-plot Micropore volume (cm^3 g^{-1})
NaUZSM-5	319	0.20	235	0.12
5Co/NaUZSM-5	299	0.22	200	0.11
10Co/NaUZSM-5	327	0.22	192	0.10
15Co/NaUZSM-5	270	0.20	146	0.07

Table S8 Surface acidity of NaUZSM-5 loaded with various metals derived from peak integration of NH_3-TPD signals.

Catalyst	Weak site acidity (mmol g^{-1})	Strong site acidity (mmol g^{-1})	Total acidity (mmol g^{-1})
NaUZSM-5	32	0	32
Co/NaUZSM-5	8	16	24
Mo/NaUZSM-5	201	0	201
Ag/NaUZSM-5	16	0	16
Ga/NaUZSM-5	35	0	35
Ce/NaUZSM-5	48	0	48

Table S9 Structural properties of Co/NaUZSM-5 and Co/NaUZSM-5-IPB.

Catalyst	BET Surface area (m^2 g^{-1})	Micropore surface area (m^2 g^{-1})	External surface area (m^2 g^{-1})
Co/NaUZSM-5	327	192	135
Co/NaUZSM-5-IPB	305	169	136

Tables S10 Detailed compositional analysis liquid products over zeolite-based and metal oxide-based catalysts for catalytic cyclohexene pyrolysis. The blank cells indicate the yield of corresponding product is below detection limit.

Selectivity* (wt%)	ZSM-5	NaZSM-5	UZSM-5	NaUZSM-5	Co/NaUZSM-5	Silica	Alumina	Zirconia	Titania
p	1.02	0.84							
2-mp	0.53	0.74							
3-mcp			8.22	10.87	10.80	7.06	10.83	9.31	10.61
4-mcp			3.49	5.90	4.99	3.83	5.49	4.92	4.98
mcpa	0.70	0.95	6.79	1.15	1.00	9.00	3.86	17.16	6.66
5-m1,3-cpde				1.16		1.12	0.48		0.98
1-mcp			45.85	71.74	79.41	46.8	71.01	44.90	55.73
b	11.61	21.23	1.01	1.13		10.96	1.80	0.75	0.83
cha	1.09	0.66	1.65	3.49	2.24	5.16	4.97	20.79	18.38
chde	0.83	0.46	6.68	0.91	1.02	1.09	0.48	1.15	1.10
t	16.27	27.32	4.37	3.64	0.55	14.98	1.08	1.01	0.73
eb	5.08	4.65	1.79						
x	20.55	24.46	4.51						
C9a	25.55	13.3	6.93						
C10a	5.96	3.71	6.65						
C11a	5.57	1.68	2.07						
C12+a	5.23								

*p=pentene, 2-mp=2-methylpentene, 3-mcp=3-methylcyclopentene, 4-mcp=4-methylcyclopentene, mcpa=methylcyclopentane, 5m-1,3-cpde=5-methyl-1,3-cyclopentadiene, 1-mcp=1-methylcyclopentene, b=benzene, cha=cyclohexane, chde=cyclohexadiene, t=toluene, eb=ethylbenzene, x=xylenes, C9a=C9 aromatics, C10a=C10 aromatics, C11a=C11 aromatics, C12+a=aromatics with 12 or more carbon atoms

Table S11 Equilibrium calculation of methylcyclopentene isomers.

Reactant	Product	ΔG(673.15 K, kJ mol^{-1})	ln$K$	K
1-mcp	3-mcp	8.0	-1.44	0.237
1-mcp	4-mcp	16.8	-2.99	0.050

Table S12 Comparison of relative content among methylcyclopentene isomers derived from thermal equilibrium calculation and experimental result.

Method	1-mcp (%)	3-mcp (%)	4-mcp (%)
Thermal equilibrium calculation	77.7	18.4	3.9
Experimental result	79.5	14.6	5.9
